# Letter to the Editor: Relations between subjective well-being and Alzheimer's disease: a systematic review

**DOI:** 10.1590/1980-57642020dn14-040018

**Published:** 2020-12

**Authors:** Vitor Maia Arca, Laiza de Oliveira Lucena, Breno José Alencar Pires Barbosa

**Affiliations:** 1 Instituto de Medicina Integral Prof. Fernando Figueira – Recife, PE, Brazil.; 2 Group of Cognitive and Behavioral Neurology, Department of Neurology, School of Medicine, Universidade de São Paulo – São Paulo, SP, Brazil.

Dear Editor,

We read with great interest the article by Moura et al. entitled “Relations between subjective well-being and Alzheimer's disease: a systematic review”.^1^ In the study, the authors performed a comprehensive systematic review of the literature to evaluate the methodological quality of studies addressing Subjective Well-Being (SWD) in Alzheimer disease (AD). Given the importance of this discussion and the fact that most neurologists may not be familiar with SWB measures (ourselves included), we missed a more detailed description of the instruments used in the articles, as well as their potential strengths and limitations. We observed that 11 different SWB measurement tools were used in the 13 articles reported. The quality of life in AD (QoL-AD) scale by Longsdon et al.^2^ was the most used tool, being reported in 5 of the 13 studies. The instrument has been translated into Portuguese and consists of 13 items related to physical health, humor, memory, task performance, interpersonal relations, and leisure activities.^3^ A review pointed out that QoL-AD was the best-researched measure, since it is available both in the self-rating and proxy-rating versions.^4^ Another point to be considered in the review by Moura et al. was the low number of results found on the PubMed platform as compared to the other searched databases. We tried to conduct the same inquiry and found a significantly higher number of studies, which led us to wonder whether other filters could have been used. We believe further studies in the field to be extremely welcome.
