# Supplementary material for: Letter to the Editor: Relations between subjective well-being and Alzheimer's disease: a systematic review
Source: Dement Neuropsychol. 2020 Dec;14(4):440–1. doi: 10.1590/1980-57642020dn14-040018 (PMC7735044; doi:10.1590/1980-57642020dn14-040018)
Supplement: Related texts

# Authors' reply: Relations between subjective well-being and Alzheimer's disease: a systematic review

**Authors:** Fernanda Panage Moura, Amer Cavalheiro Hamdan

**Affiliations:** 1 Postgraduate Program in Psychology, Universidade Federal do Paraná – Curitiba, PR, Brazil.

To the Editor,

First, we would like to thank the reader for the letter commenting on our paper.^1^ Some points can be clarified in response to the questions. The smaller number of articles found in PubMed, in relation to other platforms, is probably explained by the fact that it is aimed especially at medical studies. The term “Well-Being”, when used as a search keyword, was more frequent on platforms that cover research in other areas, such as psychology.

The search for articles was established according to previously defined inclusion and exclusion criteria, explained in the paper. On the PubMed platform, these criteria were included according to the filters shown in Table 1.

**Table 1:** Inclusion criteria and filters for the selection of articles.

Inclusion criteria	Filter	Criteria selected
Criteria A Articles with a sample of the elderly population	Age	Aged: 65+ years.
Criteria B Empirical articles	Article type	Clinical trial; controlled clinical trial; and randomized trial
Criteria C Articles published from 2014 to 2019	Results by year	01 June 01, 2014 to 01 June 01, 2019

Regarding criterion A, “Aged 65+ years” was selected. It should be noted that, on this platform, there were no other age options related to the aged population. Regarding criterion C, the search covered the last five years and was carried out in June 2019. Therefore, the filter to be used must be specified between June 2014 and June 2019.

Regarding the use of search terms, some relevant aspects need to be highlighted. The terms “Well-Being”

AND “Alzheimer” were used in the title and summary of the studies. The search result was recently reproduced and can be found at https://drive.google.com/file/d/1DYxw-6B6qZp6ZWrM1Iegnf5dMviUT64mV/view?usp=sharing

Finally, we share the same opinion regarding the concept of “Well-Being.” The same should be investigated more deeply in the different health areas. We appreciate your comments and questions regarding the article. In this way, we can improve future research and disseminate knowledge on this very current and relevant topic.

## References

[1] Moura FP, Hamdan AC (2020). Relations between subjective well-being and Alzheimer's disease: A systematic review. Dement Neuropsychol.
